# Anesthetic management for the sleep-awake-sleep technique of awake craniotomy using a novel benzodiazepine remimazolam and its antagonist flumazenil

**DOI:** 10.1186/s40981-021-00417-z

**Published:** 2021-01-31

**Authors:** Akari Yoshida, Saori Kurata, Kotaro Kida, Tsunehisa Tsubokawa

**Affiliations:** Department of Anesthesiology, School of Medicine, The Jikei University, 3-25-8 Nishi-Shimbashi, Minato-ku, Tokyo, Japan

In awake craniotomy, complete arousal and sufficient analgesia are crucial for the patient to perform the tasks. Although propofol and dexmedetomidine have been used in the past, they sometimes cause delayed recovery, excitation, and insufficient awakening [1]. Remimazolam is a novel benzodiazepine that has recently been used for clinical anesthesia in Japan and is characterized by its ultrashort-acting property with flumazenil as an antagonist. We report a case of awake craniotomy in which the patient was anesthetized with remimazolam antagonized with flumazenil.

A 48-year-old right-handed man was scheduled for awake craniotomy to prevent spatial cognitive impairment. The patient had his first generalized tonic seizure 6 weeks previously, and brain imaging revealed a 46-mm tumor in the right parietal lobe. The patient visited the operating theater before surgery, practiced the neurological assessment task, and also confirmed that the patient positioning was comfortable. On the day of surgery, anesthesia was induced with 6 mg/kg/h of remimazolam and a 100-μg remifentanil bolus, and a laryngeal mask was inserted. Supraorbital nerve block, auriculotemporal nerve block, and greater and lesser occipital nerve block were performed before skull pinning. During the initial asleep phase, the patient was artificially ventilated to control intracranial pressure with continuous infusion of remimazolam 0.75–1 mg/kg/h and remifentanil 0.1 μg/kg/min. After dural opening, remimazolam infusion was discontinued, and remifentanil was reduced to 0.03 μg/kg/min. Flumazenil was administered as a bolus of 0.3 mg when the bispectral index reached 75. The patient was awakened 3 min after flumazenil administration, and the laryngeal mask was removed. The patient was not in an agitated state, could speak, and did not complain of pain. Tumor resection was performed after confirmation of the absence of spatial cognitive dysfunction using the Raven color matrix test and the bisector test. The patient was awake for 2 h and 37 min. After tumor resection, the patient was re-anesthetized with propofol and remifentanil, and the laryngeal mask was re-inserted. After completion of surgery, propofol and remifentanil administration was discontinued, and the patient regained consciousness promptly. The overall operation time was 5 h and 22 min, and the anesthesia time was 8 h and 25 min. The postoperative interview revealed that the patient retained his memory during the awake phase, and there were no symptoms such as spatial neglect, apraxia, or paralysis. Despite the complexity of the task, the patient was able to perform it perfectly, and this anesthetic protocol was highly appreciated by the surgeons.

Sato et al. reported a case of awake craniotomy using remimazolam without flumazenil [2]. In the present case, we used flumazenil and found that it facilitated safe and quick arousal. Flumazenil has been reported to induce seizures as a side effect; therefore, the dosage should be minimized [3]. We administered 0.3 mg, and a seizure attack was observed just after cortical stimulation, which was judged to be unrelated to flumazenil. We concluded that with its ultrashort-acting property and the availability of an antagonist, remimazolam in combination with flumazenil can be a powerful tool in awake craniotomy.

## Data Availability

Not applicable.
